# Modelling morbidity for neglected tropical diseases: the long and winding road from cumulative exposure to long-term pathology

**DOI:** 10.1098/rstb.2022.0279

**Published:** 2023-10-09

**Authors:** Anna Borlase, Joaquin M. Prada, Thomas Crellen

**Affiliations:** ^1^ Department of Biology, University of Oxford, Oxford OX1 3SZ, UK; ^2^ Big Data Institute, Li Ka Shing Centre for Health Information and Discovery, University of Oxford, Oxford OX3 7LF, UK; ^3^ Faculty of Health and Medical Sciences, University of Surrey, Guildford GU2 7XH, UK; ^4^ School of Biodiversity, One Health & Veterinary Medicine, Graham Kerr Building, University of Glasgow, Glasgow G12 8QQ, UK; ^5^ Wellcome Centre for Integrative Parasitology, Sir Graeme Davies Building, University of Glasgow, Glasgow G12 8TA, UK

**Keywords:** trachoma, schistosomiasis, foodborne trematodiasis, morbidity, modelling, neglected tropical diseases

## Abstract

Reducing the morbidities caused by neglected tropical diseases (NTDs) is a central aim of ongoing disease control programmes. The broad spectrum of pathogens under the umbrella of NTDs lead to a range of negative health outcomes, from malnutrition and anaemia to organ failure, blindness and carcinogenesis. For some NTDs, the most severe clinical manifestations develop over many years of chronic or repeated infection. For these diseases, the association between infection and risk of long-term pathology is generally complex, and the impact of multiple interacting factors, such as age, co-morbidities and host immune response, is often poorly quantified. Mathematical modelling has been used for many years to gain insights into the complex processes underlying the transmission dynamics of infectious diseases; however, long-term morbidities associated with chronic or cumulative exposure are generally not incorporated into dynamic models for NTDs. Here we consider the complexities and challenges for determining the relationship between cumulative pathogen exposure and morbidity at the individual and population levels, drawing on case studies for trachoma, schistosomiasis and foodborne trematodiasis. We explore potential frameworks for explicitly incorporating long-term morbidity into NTD transmission models, and consider the insights such frameworks may bring in terms of policy-relevant projections for the elimination era.

This article is part of the theme issue ‘Challenges and opportunities in the fight against neglected tropical diseases: a decade from the London Declaration on NTDs’.

## 1. Introduction

Neglected tropical diseases (NTDs) are a diverse group of diseases which typically affect the world’s poorest and most marginalized populations [1–3]. They can cause a broad spectrum of both reversible and irreversible adverse health outcomes, or sequelae, often leading to permanent disability, with many NTDs therefore considered to be both drivers and manifestations of poverty [4]. Here we define morbidity as disease, disability or poor health and we focus on NTDs that result from infectious agents.

In contrast to the large number of infectious agents where the range of potential sequelae are observed within a relatively short time-frame after infection, the morbidity attributable to some NTDs can be long-term in nature and arise over many years of chronic or repeated infection [5]. This is particularly true for pathogens where individuals do not develop protective immunity to infection, and where morbidity risk is generally thought to increase as a function of cumulative parasite burden (macroparasites) [6] or repeated re-infection (microparasites) [7–9]. This draws comparisons with non-communicable diseases where a ‘dose–response’ effect between exposure to a risk factor and clinical outcome has been demonstrated, with smoking and lung cancer one of the most well characterized examples. Yet, as with non-communicable diseases, there are considerable heterogeneities in NTD-attributable morbidity outcomes between individuals with a comparable exposure, suggesting a role for host-specific factors, such as individual immune response, genetic predisposition and co-morbidities, which remain in most cases poorly elucidated [10–12].

In recent years, coordinated global efforts have seen significant and unprecedented advances have been made towards NTD control and elimination targets set by the World Health Organization (WHO) for 2030 [3]. However, epidemiological thresholds have often been determined based on broad assumptions regarding the relationship between prevalence of infection and the resulting burden of disease [13]. At low levels of transmission, and particularly near elimination, stochastic processes and individual-level heterogeneities become more important, determining whether infection dies out, is maintained at a low level or resurges [14]. Such heterogeneities are also critical to determining morbidity outcomes at the individual and population level. Predicting how prevalence and the distribution of pathology resulting from NTDs may change as control programmes reduce transmission through public health interventions is a key operational and policy challenge for NTDs [15].

Mathematical models of infectious diseases that incorporate morbidity outcomes have been used for many years to inform public health policy. For some NTDs, the relationship between exposure and risk of morbidity (and/or mortality) is relatively well quantified, which has facilitated explicit incorporation of morbidity outcomes into dynamic transmission models. Examples where the time-lag between infection and severe sequelae is relatively short, and timely detection and treatment of clinical cases is critical to reducing morbidity (and mortality), include visceral leishmaniasis (VL) and human African trypanosomiasis (HAT). In both these cases, models have been able to capture the morbidity impact of different interventions, including those that aim to reduce transmission, as well as alternative screening and case detection protocols. Such models have provided key insights of relevance for policy and planning for NTD programmes, including identifying regions that may need more intensive interventions [16] and evaluating the likely effectiveness of WHO-recommended protocols in terms of reaching elimination targets [17], and by defining the utility of interventions in terms of disability-adjusted life-years (DALYs), models have also been used to quantify cost-effectiveness of programmes [18]. By contrast, leprosy is an example of a disease where the time to onset of symptoms for the most severe form (multibacillary leprosy) is prolonged (more than 10 years). Nevertheless, transmission models of leprosy have been able to use relatively simple assumptions regarding the proportion of population that is susceptible and probability of different clinical forms of leprosy to provide policy-relevant insights regarding the impact of interventions on the distribution of morbidity outcomes [19,20].

For those NTDs where morbidity risk is associated with increasing cumulative exposure, the relationships between infection dynamics and long-term disease are generally more complex at both the individual and population level, in addition to a temporal disconnect between infection and severe sequelae that may be years or decades. These factors, together with heterogeneities in both host exposure and host response to infection, present significant challenges to incorporating morbidity outcomes into transmission models. An important example of an NTD where this complex modelling challenge has largely been addressed is onchocerciasis, where both population-based deterministic models [21] and individual-based models [22–24] have been developed that incorporate a range of morbidity outcomes as a function of cumulative microfilariae exposure. These models have generated projections that encompass long-term morbidity trends after mass treatment as well as the cost-effectiveness of programmes (in terms of DALYs averted) for a range of transmission settings, providing quantitative insights of significant value for onchocerciasis programmes. However for some NTDs where long-term morbidity is associated with cumulative exposure, significant gaps remain in our understanding of how prevalence, incidence, and distribution of chronic pathology may change after implementation of control programmes, and many currently used models do not explicitly include long-term morbidity [25,26].

In this perspective, we consider how epidemiological and clinical data applied to analytical frameworks and transmission models may seek to improve our understanding of how morbidity risk associated with cumulative exposure is distributed in populations before and after interventions, including projections of future morbidity trends. Focusing on frameworks applicable to those NTDs where significant modelling gaps remain, we build on concepts proposed by Medley *et al.* [6,27], using case studies of trachoma, schistosomiasis and foodborne trematodes. We consider empirical data needs and identify priority questions of relevance to public health policy, advocacy and resource allocation which future models may seek to address, questions of increasing relevance if programmes are to truly deliver on promises to relieve the burden of morbidity from NTDs [1,28].

## 2. Key challenges

### (a) A dose–response relationship? Cumulative damage and risk of long–term morbidity in neglected tropical diseases

The relationship between cumulative exposure and long-term sequelae for NTDs draws comparisons with toxicology and non-communicable diseases (NCDs), where a dose–response relationship is often demonstrated between an increasing level of exposure and probability of clinical outcome [29]. Cigarette smoking is one of the most well characterized examples, for which a large number of studies have been able to describe the dose–response relationship in terms of smoking intensity, smoking duration, pack-years and years since cessation of smoking for a range of outcomes, including cancers and stroke [30–32].

**Figure 1.**
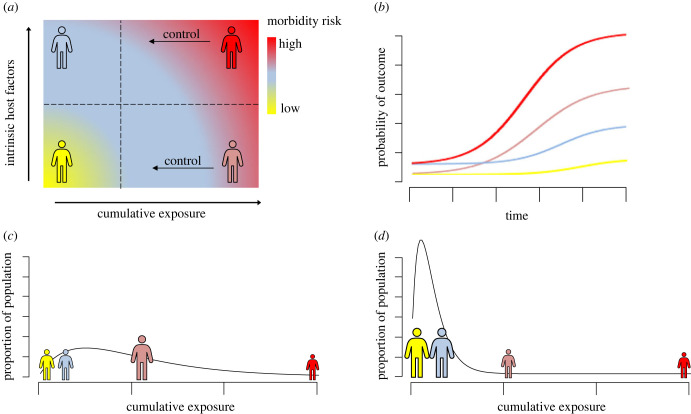
The interaction between cumulative exposure and intrinsic host factors (such as genetic predisposition) in determining morbidity risk (*a*) and how differences in morbidity outcome may be observed over time in exposed populations (*b*): individuals with low intrinsic risk and low exposure (yellow); high intrinsic risk and low exposure (blue); low intrinsic risk and high exposure (pink) and, high intrinsic risk and high exposure (red). The shift in distribution of morbidity risk from before an intervention (*c*) to after an intervention (*d*), demonstrates how heterogeneities in intrinsic risk factors and exposure may become more important as we move towards elimination.

**Figure 2.**
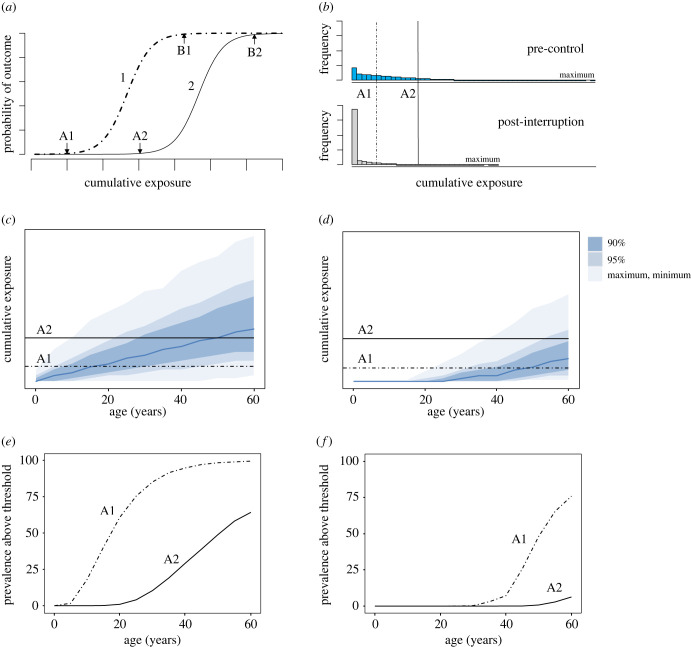
(*a*) Example dose–response relationship for two morbidity outcomes. (*b*–*f*) Output from an agent-based model that tracks lifetime history of exposure, where interruption of transmission is simulated and the population tracked for 20 years, showing how morbidity risk as a function of the population above given thresholds may be distributed in the whole population (*b*) and by age, before (*c*,*e*) and after (*d*,*f*) interruption of transmission. (*c*) Pre-control, (*d*) post-interruption, (*e*) pre-control, (*f*) post-interruption.

Dose–response relationships are often described using a sigmoid function analagous to those shown in figures 1*b* and 2*a*. Often there is a threshold below which the outcome will not be observed (A1 and A2 in figure 2*a*), or a background level of morbidity not associated with exposure (the *y*-intercept in figure 1*b*), with the slope of the curve then related to the strength of the dose–response relationship above these values. Analyses of low-dose smoking, including passive smoking, have found that there is no limit below which the risk of adverse health outcome does not increase (no minimum ‘threshold’), and that 100% smoke-free environments are the only way to adequately protect people from the harmful effects of tobacco smoke [33].

Characterizing the relationship between exposure and risk of different clinical outcomes is key to making morbidity projections for NTDs. For morbidities associated with cumulative exposure this includes establishing if there is a threshold of exposure below which these outcomes will not be seen, or if, as with smoking, for certain NTDs the only way to protect people will be to eliminate exposure entirely by interruption of transmission.

Parameterizing the functional form for the dose–response relationship for NTD morbidities associated with cumulative exposure is, however, complicated by challenges in gathering data that capture the history of exposure at the individual level. Self-reporting of intensity and duration of smoking is generally used in studies of smoking exposure, and for some parasitic diseases, such as cysticercosis, analysis of experimental infections in animals with known exposures has enabled quantification of the dose–response relationship for a range of clinical outcomes [34]. Neither self-reported exposure nor precise infectious dose data are practicable for most NTDs, but in some cases indirect measures of infection intensity can be used to infer infection history. One example is onchocerciasis (river blindness), where modelling frameworks combined with long-term, individual-level data (from the Onchocerciasis Control Program in West Africa between 1974 and 2002) that included proxy metrics for intensity of infection (microfilarial density in skin snips) enabled characterization of the relationship between exposure to the helminth *Onchocerca volvulus* (measured as cumulative adult worms or density of microfilariae) and the probability of the resulting blindness (figure 4*a*) [35–37]. However, cohort studies that capture such individual-level data are generally costly and therefore not commonly incorporated into NTD surveillance. An alternative approach is calibrating individual-based models (which track age and cumulative exposure) to reproduce population-based data on infection prevalence and prevalence of morbidity, which can allow the relationship between exposure (generally a function of age in endemic settings) and morbidity to be inferred. This has been recently demonstrated for onchocerciasis [23,24], where age-group specified population-based data were used to parameterize individual-based models that track accumulation (and also regression) of tissue damage due to dying microfilariae. These models have then been used to predict a range of morbidity outcomes (both acute/reversible and chronic/irreversible) as a function of this accumulated tissue damage.

One of the challenges in proving the link between tobacco and cancer in the 1950s was that a high proportion of the population in the UK and the USA (where most early studies were performed) were smokers but only a fraction of these would develop lung cancer, while conversely a small number of non-smokers acquired lung cancer [38]. Today the role of individual host genetic polymorphisms in determining risk of acquiring lung cancer for both smokers and non-smokers is increasingly well quantified [39,40]. For example, a large cohort study in which polygenic risk scores were calculated based on known genetic variants associated with lung cancer found that the strength of the association between smoking and lung cancer increases with increasing genetic risk, with the greatest risk in high genetic risk score individuals with the highest smoking pack-years [32].

This interaction between host-specific risk factors and exposure in determining risk of morbidity is represented schematically in figure 1*a*. Here, the lowest morbidity risk is in individuals with low individual risk factors and low exposure (for example, a non-smoker with low genetic risk of lung cancer), and the highest risk is in individuals with high exposure and high individual risk factors (heavy smoker with high genetic risk of lung cancer). Figure 1*b* shows how this may translate to different probabilities of the outcome over time, with the *y*-intercept (representing the baseline risk for the unexposed population) analogous to the non-smoker population in the smoking example, and the difference between the low genetic risk group and high genetic risk individuals being exaggerated as exposure is increased over time.

Similarly for many NTDs, increased exposure over time leads to increased risk of outcome, and thus reducing the rate of exposure will reduce overall morbidity risk in a population (figure 1*a*). In most cases, the ultimate aim of NTD control programmes is interruption of transmission (which will effectively arrest cumulative exposure in a population, discussed in §2b). However, in some cases, for example, schistosomiasis, WHO definitions of ‘morbidity control’ or ‘elimination as a public health problem’ do not correspond to interruption of transmission, and whilst transmission persists, accumulation of exposure will continue, even if at a reduced rate. In many cases, it is unclear if at low levels of exposure, there will still be significant risk of long-term NTD-associated morbidity, in particular for individuals with high individual risk factors. Such host-specific factors have been suggested to comprise immune response [41], genetic predisposition [42] and co-morbidities [43]. Incorporating variability in host susceptibility to morbidity for a given level of exposure is critical to ensuring realistic morbidity projections; however, the role of individual host factors in determining morbidity outcomes is often poorly understood for NTDs. Nevertheless, even where the precise mechanism for variations in clinical outcomes is poorly characterized, parameters that represent this heterogeneity can be estimated by fitting individual-based models to age-specified morbidity data from a range of transmission settings, as has been demonstrated for onchocerciasis [23,24].

### (b) Shifting or reshaping the curve? Exposure and morbidity risk before and after interventions

For many NTDs, exposure within and between endemic populations is highly heterogeneous, contributing to the observed heterogeneity in morbidity outcomes at the individual and population level (i.e. individual risk of different morbidity outcomes and proportion of individuals with specific outcomes in different populations).

For helminths, the variability in exposure typically leads to overdispersion in individual worm burden, often represented using a negative binomial distribution; the worm burden in an individual is drawn from distribution NB(*M*, *k*), where *M* is the mean worm burden in the population and *k* describes the degree of dispersion [44,45]. Here, a small *k* represents overdispersion (i.e. most people have few worms, a few people have a large number of worms), with increasing *k* moving towards a Poisson distribution corresponding to worms being randomly distributed within the population.

Quantifying how distribution of exposure risk in a population may change over the course of and after an intervention, and establishing the impact this will have on prevalence and incidence of clinical outcomes given the distribution of individual host factors in the population are critical for projecting long-term morbidity outcomes. This is represented schematically in figure 1*c*,*d*. In a pre-control scenario with an overdispersed exposure (low *k*), the majority of people will have high enough exposure that they are at risk of morbidity (pink), with a small number of the most at-risk people having the highest risk of morbidity (red) (figure 1*c*). The exposure curve may, however, be reshaped after an intervention (figure 1*d*, where the mean exposure is decreased compared with figure 1*c*). Here, the overall morbidity risk in the population is decreased as most people have low exposure, and so the impact of individual risk factors (i.e. the difference in risk between yellow and blue) may become more important, as will identifying any subsets of the population that remain at high risk (red). This picture would be exaggerated if there was significant systematic non-adherence to an intervention (e.g. subsets of the population who do not participate in interventions, such as mass drug administration (MDA)), which may increase heterogeneity in exposure after an intervention (decreased *k*).

Where pathological processes have a long time-course, the impact of accumulated damage from infections may not be apparent for many years, presenting a further challenge for projecting future morbidity burden in a population. Studies of outcomes for smokers and former smokers show that whilst for every year of smoking risk of disease rises, for every year of abstinence risk decreases, essentially linear curves with opposite but similar slopes [31]. If smoking ceases completely in a population with a heavy prior usage of cigarettes, future cases of smoking-induced lung cancer would still be expected as the population ages, as while individuals would be at decreased risk at a given age depending on age at smoking cessation, figure 4*c*, previous damage from the chemical carcinogens in tobacco will have set some individuals on an irreversible path to tumorigenesis [46].

Similarly for NTD morbidities with long time-courses, even if transmission is interrupted, the consequences of accumulated damage may play out in terms of incident cases for many years as the exposed population ages. How many incident cases will be seen and for how long will be determined both by the level of endemicity before interruption of transmission, how long it took for this to be achieved, and the demographic turnover of the population. This is represented by the example shown in figure 2, which shows the output from an individual-based transmission model that tracks age and cumulative lifetime exposure of individuals to infections (based on the model described in [26]). In this example, interruption of transmission is simulated and the population is then tracked for 20 years. Example dose–response curves are shown for two different forms of morbidity (1 and 2), figure 2*a*, with A1 and A2, respectively, representing the thresholds below which we do not expect to observe infection-related morbidity. In endemic settings, average cumulative exposure will increase over time (i.e. with the age of individuals), and thus older individuals are more likely to have lifetime exposure above these thresholds and therefore are at higher risk of morbidity, both before and after interventions. The frequency distribution of cumulative exposure in this example population before control and 20 years after interruption of transmission can be compared, figure 2*b*. Owing to population turnover (natural attrition of older individuals and birth of new individuals who are not exposed), the cumulative exposure curve is reshaped and 20 years post-interruption most individuals are now below the threshold for morbidity. However, these histograms do not capture the underlying cohort effect, with figure 2*c*–*f* showing age-specified output from the same model, highlighting the limitations of surveillance strategies that focus on prevalence of infection or markers of recent infection in younger populations only. Age-specified cumulative exposure is shown before control, figure 2*c* and 20 years after interruption of transmission, figure 2*d*, with exposure thresholds for disease morbidity outcomes 1 and 2 shown as horizontal lines. The proportion of the population above these lines represents the population with these threshold levels of cumulative exposure or higher, which can be observed by the distribution (by age) of morbidity risk in the population before and 20 years after interruption, figure 2*e*,*f*. It can be seen that the age-specified cumulative exposure curve after interruption is shifted to the right, and while the overall morbidity risk in the population is decreased, it is now concentrated in the older population. The implication of this is that historical exposure could lead to incident cases of NTD-attributable morbidity, potentially many years after transmission has been interrupted, particularly if age is a covariate for morbidity, as is the case for NTD-associated cancers.

This emphasizes the importance of establishing how long morbidity may take to develop and if there are thresholds below which morbidity will not be seen for different types of adverse health outcome. In this general example, if we assume outcome 2, associated with higher exposure, represents a more severe pathology than outcome 1, it can be seen that post-control outcome 1 may come to represent a more significant proportion of burden of morbidity, as whilst outcome 2 may be more severe, there may be almost no cases, figure 2*f*.

**Figure 3.**
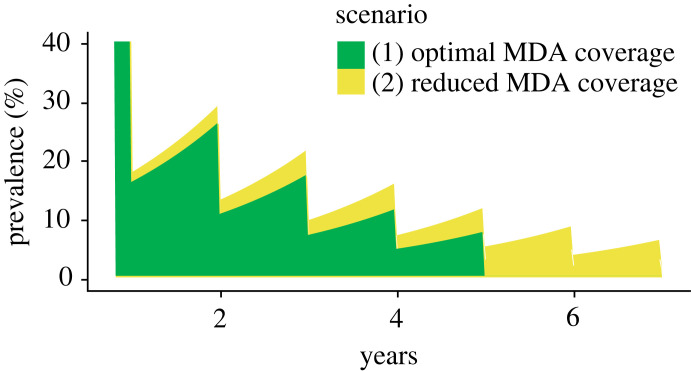
The potential impact of delays to achieving elimination in terms of excess morbidity risk, with area under the curve representing burden of infections in a population where (1) interruption of transmission is achieved after 5 years of mass drug administration (MDA) and (2) interruption of transmission takes 7 years of MDA owing to sub-optimal coverage.

The time course over which control is achieved will also impact the degree of accumulated damage in a population and therefore future morbidity. Figure 3 shows a simple resurgence model where prevalence of infection is assumed to increase exponentially after yearly MDA until transmission is interrupted for two scenarios: (1) optimal MDA, where interruption of transmission is achieved after 5 years; (2) reduced MDA coverage, which means it takes 7 years for transmission to be interrupted. If the area under the curve can be considered representative of the cumulative burden of infections it can be seen that the less effective programme, which takes longer to interrupt transmission, will correspond to greater overall cumulative burden owing to both increased levels of infection for the first 5 years, and then two additional years before control is achieved.

These general examples illustrate how, for diseases with long-term morbidity outcomes, those settings where pre-intervention transmission was higher and achieving control was slower may need support for managing NTD-associated morbidity for a longer time-frame even after prevalence goals are achieved. Mathematical modelling can play a role in identifying where and for how long post-control morbidity management may be needed, in particular agent-based models that track cumulative exposure at the individual level [23,24].

## 3. An epidemiological framework for neglected tropical diseases

Evidence for many helminth species supports the hypothesis that parasite-induced morbidity is primarily due to cumulative exposure over an individual’s lifetime [6,47]. Consequently the presence of the pathogen does not necessarily indicate presence of the disease (which may arise decades later) and, conversely, presence of the disease may occur at a later time when a person is no longer infected with the pathogen. Traditional epidemiological approaches to identifying and quantifying associations between infection status and the presence of morbidity, such as logistic regression, would therefore be inappropriate. Instead it is important to consider the dynamic process that gives rise to morbidity over time. While longitudinal studies that track individuals over time are often costly or impractical, age-specified cross-sectional surveys can provide crucial insights. Considering that time and age are related variables, data can be analysed using ‘force of infection’ models (also known as ‘catalytic curves’). An implication for analysing epidemiological surveys is that, in the case of chronic exposures and outcomes, a person’s age is not simply another fixed effect covariate to be included in a regression model but should be considered separately as age is linked to the duration of exposure.

We present here a simplified modelling framework to elucidate the key aspects of macroparasite transmission relevant for morbidity. The mean number of adult worms within a population *M* changes with respect to host age *a* in years and time in years *t*, owing to the force of infection Λ(a,t) and the spontaneous mortality rate of adult worms *μ* [44],

3.1 $$\frac{\partial M(a,t)}{\partial a}+\frac{\partial M(a,t)}{\partial t}=\Lambda (a,t)-\mu M(a,t).$$

Heterogeneity in exposure is introduced into the model with the term *s*_*i*_ for each person within the population; this parameter follows a gamma distribution with a mean of 1 and shape parameter *k*, which describes the degree of dispersion [48]. Each individual is therefore subject to a force of infection

3.2 $$\Lambda_{i}(a,t)=s_{i}\Lambda (a,\;t).$$

The nature of the *s*_*i*_ term will vary by NTD according to the route of transmission. For vector-borne parasites it could be linked to a person’s biting rate, or for foodborne pathogens to a ‘feeding rate’. From equation (3.1), each individual has an expected worm burden *M*_*i*_(*a*, *t*). To allow for an integer number of worms per-person, *M*_*i*_(*a*, *t*), which is a continuous variable, can be thought of as the rate parameter for a Poisson sampling process that gives rise to the true number of worms per person *x*_*i*_(*a*, *t*). This gamma-distributed susceptibility in the population, combined with a Poisson sampling process, is equivalent to a negative binomial distribution [49,50], which is commonly used to describe distribution of parasite burden in a community [51].

Our framework assumes that the pathological damage for each person due to the parasite, denoted *D*_*i*_(*a*), is proportional to the cumulative parasite burden: *D*_*i*_(*a*) ∝ *w*_*i*_(*a*) [6]. We obtain the cumulative worm burden at age *a* for an individual *i*

3.3 $$w_{i}(a)=\int_{0}^{a}M_{i}(a^{\prime },t)\;da^{\prime }.$$

The relationship between cumulative worm burden and the damage acquired depends on whether the helminth-induced pathology (i) resolves following a cessation in infection [52], for example, after treatment, or (ii) continues to progress in the absence of additional infections, as is the case for carcinogenesis following a critical driver mutation in a proto-oncogene or tumour suppressor gene.

The probability of developing a specific morbidity for person *i* within the population is given as a function of the cumulative worm burden *w* with set of parameters *θ*, where *Q*_*i*_(*a*) indicates the disease state of person *i* at age *a* for a given condition (1 if positive, 0 if negative),

3.4 $$\mathrm{Pr}(Q_{i}(a)=1)=f(w(a)|\;\theta ).$$

The simplest function assumes that parasite-induced damage does not resolve (*D*(*a*) = *w*(*a*)) and there is an individual threshold *ξ*_*i*_ for the onset of morbidity given by the cumulative worm burden, and the probability is 50% when *w*_*i*_(*a*) = *ξ*_*i*_ [37], for instance, a logistic function [49]

3.5 $$\mathrm{Pr}(Q_{i}(a)=1)=\frac{1}{1+e^{-(w_{i}(a)-\xi_{i})}}.$$

A more complex formulation allows the probability of disease status to change dynamically and resolve following treatment [6,53]. The accumulated damage *D*_*i*_(*a*) is therefore

3.6 $$\frac{dD_{i}}{da}=M_{i}(a)-\gamma ,$$

where *γ* is a constant rate of recovery from tissue damage, as has been incorporated into models for schistosomiasis [6] and onchocerciasis [24]. Therefore, the probability of sequela changes as a function of damage

3.7 $$\mathrm{Pr}(Q_{i}(a)=1)=\frac{1}{1+e^{-(D_{i}(a)-\xi_{i})}}.$$

Given the complexities of estimating morbidity risk at the individual level, a common approach to projecting the burden is to relate the observed prevalence for a given helminth at the community level with the prevalence of morbidity [54–56], as this can be achieved using population-level cross-sectional survey data, which may be more readily available than individual-level data. This non-mechanistic approach has a number of limitations, however, as it fails to account for the effect of host age, the time-dependent nature of disease incidence, and heterogeneity in exposure between infected individuals (as infection intensity is not captured by prevalence). If pathogen transmission has recently increased or decreased sharply, the lag between changes to transmission and the development of chronic pathology could lead to prevalence of the infection and prevalence of long-term sequelae appearing uncorrelated in a simple regression analysis (as described in §2b). Assuming a negative binomial distribution of adult worms within the host population, the parasite prevalence at age *a*, *p*(*a*), can be expressed as function of the mean number of mature parasites *M*(*a*) and the aggregation parameter *k*,

3.8 $$p(a)=1-(1+\frac{M(a)}{k}{)}^{-k}.$$

The true population parasite prevalence *p* is thus

3.9 $$p=\frac{\sum_{a=0}^{\infty }p(a)N(a)}{N},$$

where *N*(*a*) is the number of hosts with age *a* and *N* gives the overall population size. Given imperfect diagnostics, the true prevalence is related to the observed prevalence *p*′ as

3.10 $$p^{\prime }=p\mathrm{se}+(1-p)(1-\mathrm{sp}),$$

where se and sp are, respectively, the diagnostic test sensitivity and specificity [57]. A curve is then fitted between the observed prevalence of parasite infection *p*′ and the prevalence of the disease outcome of interest *q*, where

3.11 $$q=\frac{\sum_{i=1}^{N}Q_{i}}{N}.$$

Different functions have been proposed to model the relationship between parasite prevalence *p* and disease prevalence *q*. A study on morbidity from schistosomiasis by van der Werf *et al.* used a modified logistic expression [58]

3.12 $$q=\frac{\alpha bp^{\prime c}}{1+bp^{\prime c}}.$$

**Figure 4.**
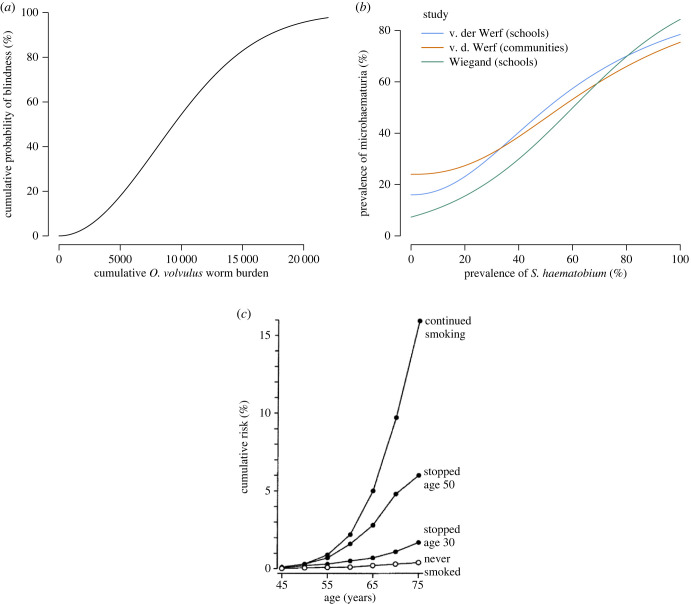
Dose–response relationships between exposures and clinical outcomes. (*a*) Relationship between cumulative *Onchocerca volvulus* worm burden and cumulative probability of blindness used in the ONCHOSIM agent-based model [37]. (*b*) An example of an ecological, or population-level, dose–response between community prevalence of *Schistosoma haematobium* and microhaematuria (blood in urine observed under a microscope) from van der Werf & de Vlas [59] and Wiegand *et al.* [60]. (*c*) Non-communicable disease example showing the effect of stopping cigarette smoking at different ages and the cumulative risk of death from lung cancer in the UK (adapted from Vineis *et al.* [46]).

This can create a sigmoid curve similar to the dose–response curves described in §2, but where the *x*-axis can be interpreted as probability of exposure rather than level of exposure, and *α* is the baseline disease risk in the absence of parasite infection (the *y*-intercept), with *b* and *c* then parameters to be estimated. One example is the association between the prevalence of *Schistosoma haematobium* and haematuria (blood in urine) as a result of inflammation in the urinary tract from parasite eggs. The modelled results from two meta-analyses [59,60] are shown in figure 4*b*. However, the relatively high prevalence of haematuria at very low or zero observed infection prevalence is likely at least in part to be attributable to low sensitivity of parasitological diagnostics. Sensitivity of diagnostics based on egg detection is particularly poor in low worm-burden individuals [45], and studies of alternative diagnostics for schistosomiasis have indicated that prevalence post-treatment may rebound to almost the same level as pre-treatment, but with a much lower mean worm burden [61]. This highlights both the data challenges and also the limitations of using simple prevalence frameworks to make projections regarding morbidity risk populations after an intervention.

## 4. Case studies

### (a) Trachoma

Trachoma is an infectious cause of blindness, where the pathological process that ultimately leads to sight loss can be considered to take place in two phases [62]. Firstly, recurrent infection with conjunctival strains of *Chlamydia* *trachomatis* bacteria causes inflammation of the cornea and conjunctiva, and repeated rounds of severe inflammation lead to scarring of the conjunctiva, known as trachomatous scarring (TS). This scarring instigates the second phase, in which the eyelid starts to turn in (entropion), causing eyelashes to contact the surface of the eye (trachomatous trichiasis, TT) resulting in pain, and over many years, corneal scarring, corneal opacity (CO) and ultimately permanent blindness. In common with most NTDs, trachoma is found at highest prevalence in the poorest communities of low-income countries, and in 2021 it was estimated that 136 million people lived in trachoma-endemic communities, and 1.8 million people had trichiasis due to trachoma, the majority of these in sub-Saharan Africa [63,64].

WHO define the active trachoma threshold for elimination of trachoma as a public health problem to be prevalence of trachomatous inflammation-follicular (TF) in children aged 1–9 years of less than 5%, with TF a marker of recent infection. An additional criterion for elimination of trachoma as a public health problem is prevalence of trachomatous trichiasis (TT) ‘unknown to the health system’ of less than 0.2% in individuals over the age of 15 years. The twofold criteria for elimination largely reflect this need to consider management of the infection and latter disease separately, and delivering surgery to correct entropion in patients affected by TT is a crucial part of trachoma management programmes.

#### (i) From exposure to outcome: the dose–response relationship and key heterogeneities before and after interventions

**Figure 5.**
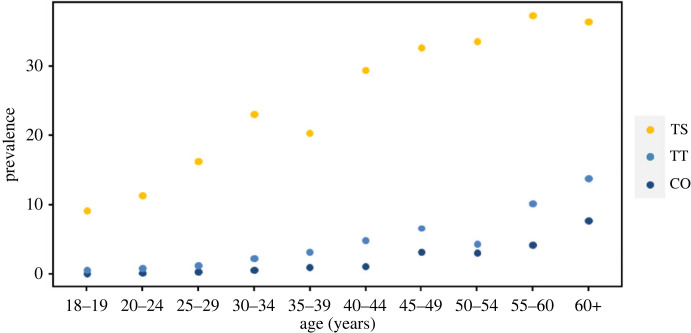
Age-specified prevalence of three progressive stages of trachoma-associated morbidity: trachomatous scarring (TS), which progresses to trachomatous trichiasis (TT), which in turn can lead to corneal opacity (CO) and permanent blindness. Data from [65].

In endemic communities first infections generally occur in early childhood, with age and lifetime number of infections strongly correlated. An increasing prevalence of both trachomatous scarring and late-stage sequelae (TT and CO) with increasing age is well documented [65–67] (illustrated in figure 5), supporting an assumption of a cumulative effect analogous to the dose–response relationship. Several prospective studies have also consistently identified a strong relationship between number of observed episodes of intense clinical inflammation and development of trachomatous scarring [67–70].

Transmission is thought to be predominantly via close contact, fomites such as shared bedding and the eye-seeking fly *Musca sorbens*. Spatially, infection and active trachoma are very focal, and studies have identified clustering at the community, neighbourhood, compound, household and bedroom levels [71–73], with crowded sleeping arrangements that favour transmission also being correlated with relative poverty. The association with poverty further complicates the picture of risk distribution, with sight loss in this context both a cause and effect of poverty, and with co-morbidities such as non-chlamydial bacterial and viral infections that potentially exacerbate inflammation also associated with overcrowding, and with unknown roles for factors such as malnutrition [74]. Women are more at risk of TT, and this gender disparity is attributed primarily to women being in closer contact with children, who form the core transmission group [75], although more biological causes for the observed sex-linked differences in outcome have not been ruled out [76].

It is known that people can suffer repeated infections, and that the rate of bacterial clearance increases and duration of inflammation decreases with age, attributable at least in part to an acquired, but not fully protective, immune response [77,78]. In ocular trachoma, it is thought that the immune response itself is immunopathological, causing the scarring that is a precursor for subsequent pathology [79]. A recent review only found two studies that had examined the association between ongoing infection and development or progression of scarring, and neither had identified a relationship after adjusting for clinical inflammation [62]. These data suggest that it may not just be the number of infections, but the type and intensity of immune response that may determine risk of scarring and progression, and it is likely that this will vary between individuals, potentially determined by age and genetic factors as well as history of infection.

#### (ii) Modelling morbidity

Given that there may be a gap of decades between markers of recent infection, such as TF, and the later stages such as TT, there is a current need to evaluate how formerly endemic communities may experience morbidity associated with trachoma in the years after the TF 5% threshold has been achieved. The distribution of lifetime number of infections and other factors relating to risk of blindness from trachoma in a community at the time WHO thresholds have been met is likely to vary depending on pre-control level of endemicity, and how long it took for an intervention to reduce prevalence, presenting challenges for planning future surgical needs in trachoma-endemic settings and targeting interventions to limit disease progression before it reaches the permanently blinding stages of CO.

Mathematical modelling could potentially play a role in answering questions such as how long after reaching the TF 5% target would surgery or other interventions be needed, and which communities are likely to need ongoing interventions for longest. These questions have increasing operational relevance as a growing number of trachoma programmes reach WHO targets [80].

Given that people can suffer repeated infections and the assumption that risk of TS and TT increases with increasing number of infections, the framework described in §3 can be adapted to trachoma. If *M*(*a*) is given to represent the average bacterial load with age, and we assume that *M*(*a*) is proportional to the average number of infections in a population, then equations (3.1)–(3.3) are applicable, with the risk of morbidity for an individual *i* at age *a* being a function of cumulative number of infections *w*_*i*_(*a*) (equation (3.3)).

The force of infection $\Lambda_{i}(a,t)$, which fundamentally determines average number of infections, and therefore average risk of morbidity at a given age, is likely to vary considerably between communities, with further heterogeneities in both exposure and individual risk factors within a community, for example, variations in immune response. However, unlike helminth infections, there are no proxy measurements for exposure that may enable quantification of an overdispersion parameter *k*, and therefore quantifying the heterogeneity in individual exposure *s*_*i*_ (equation (3.2)) is a key challenge. Biases in non-compliance also mean heterogeneity in exposure may increase after a programme of interventions. The extreme outcome of such heterogeneity would be transmission and therefore risk of blindness persisting in subsets of populations even after WHO targets have been met, as these targets do not necessarily directly correspond to interruption of transmission. Encouragingly, some studies have indicated that non-participation in MDA is associated with a lower likelihood of trachoma infection [81]; however, detailed studies of rate, distribution and factors that determine non-participation in trachoma programmes are lacking for most endemic countries.

Once TT has developed, the progression towards corneal opacity and therefore permanent blindness is an autonomous process independent of additional infection. Largely for this reason, model projections that encompass incident TT are likely to be the most informative for trachoma programmes, as TT can be corrected surgically. Furthermore, owing to intense pain and blepharospasm (involuntary spasm of muscles around the eye associated with conditions such as trichiasis), TT cases are often functionally blind, and therefore the morbidity burden at this stage may be almost equivalent to the CO stage. A significant complication for projecting long-term risk of incident TT after interventions is uncertainty regarding the degree to which conjunctival scar contraction which gives rise to TT may continue to progress in the absence of additional infection. At the population level, scarring has been observed to develop and progress over time, even after the prevalence of clinical signs of active trachoma have declined [66,69,82]. However, at the individual level, whether there is a threshold number of infections and/or level of scarring that will lead to disease progression in the absence of further infection is currently unclear, confounded by the correlation between age and infection in endemic communities and the likelihood of ageing as an independent covariate for disease progression. Using the example given in figure 2, if cumulative exposure is given as lifetime number of infections, TS is given to be morbidity outcome 1, and TT outcome 2, it is not clear if TS will continue to progress in individuals who have a lifetime number of infections greater than threshold A1 in the absence of further infections, or if incident TT will only be seen in individuals who already have a lifetime number of infections greater than A2 at the time transmission is interrupted. Figure 2*d*,*f* shows how the post-elimination risk profile for TT could vary substantially depending on assumptions regarding disease progression in the absence (A1) or the presence (A2) of additional infection.

Models have previously attempted to attribute a necessary lifetime number of infections for TS (100) and TT (150) [7], assuming a threshold above which morbidity outcome is inevitable (analogous to thresholds B1 and B2 in figure 2), but not allowing for heterogeneity in exposure or individual risk factors such as immune response and age. Further analysis of how risk factors for TS and TT are distributed in populations, cohort studies and fitting pre-and post-control age profiles of TS and TT to agent-based models may enable models to fully incorporate risk of TS and progression to TT to make more detailed projections for post-control scenarios [83].

### (b) Schistosomiasis

Schistosomiasis is caused by parasites of the *Schistosoma* genus, which have a complex life cycle mediated by a freshwater snail intermediate host. Adult worms live in the blood vessels of human hosts, and the majority of morbidity is caused by immune-mediated responses associated with parasite eggs trapped in host capillaries and tissues. In the case of urogenital schistosomiasis, caused by *S. haematobium*, this causes haematuria (blood in the urine) and a range of long-term pathologies, including fibrosis and calcification of the urinary tract, obstruction of urine flow, genital schistosomiasis, and, rarely, bladder cancer [84–86]. In the case of intestinal schistosomiasis, in Africa primarily caused by *Schistosoma mansoni*, and in Asia primarily caused by *Schistosoma japonicum*, infection causes acute morbidities such as abdominal pain, blood in faeces, diarrhoea and anaemia, and long-term pathologies include periportal fibrosis, which can lead to portal hypertension, hepatosplenic disease and in extreme cases fatal haemorrhages [84,87]. The prevalence of schistosomiasis in sub-Saharan Africa is estimated to have declined from 23.0% in 2010 to 9.6% in 2019, which is equivalent to infection in 27.6 million school-aged children [88].

The aim of current public health strategies for schistosomiasis is to decrease morbidity through periodic MDA of praziquantel, primarily to school-aged children. WHO defines morbidity control for schistosomiasis as less than 5% prevalence of heavy-intensity infections, and elimination as a public health problem (EPHP) is defined as reaching less than 1% prevalence of heavy-intensity infections (PHI) [3]. Here, heavy-intensity infections are defined as more than 50 eggs per 10 ml of urine for urogenital schisotosomiasis and more than 400 eggs per gram of stool for intestinal schistosomiasis.

#### (i) From exposure to outcome: the dose–response relationship and key heterogeneities before and after interventions

The majority of pathology associated with schistosome infections is not due to the worms themselves but immunopathological reactions to schistosome eggs trapped in host tissue [84]. The organ-specific clinical symptoms in schistosomiasis often correlate positively with the intensity of infection, demonstrated in human and animal studies, with egg count in faeces or urine used as an indirect measure of worm burden in humans. Three distinct stages of schistosomiasis have been identified: acute, established and late chronic [89]. The acute form is more commonly seen in travellers to endemic areas, but this acute form is much rarer in endemic communities, generally assumed to be due to chronic exposure [90]. The majority of schistosomiasis infections in endemic communities lead to established active infections, in which parasite eggs elicit inflammation, leading to formation of granulomas, which are progressively replaced by fibrotic deposits in chronic and advanced disease. Animal studies indicate that chronic persistent infection downregulates immune responses and limits immunopathology [91–93], and there is evidence that individuals of comparable age and infection intensities will experience differing degrees of pathology owing to innate hyperresponsiveness of some to egg antigens [94]. Granuloma formation is CD4+ T cell-dependent and is controlled via a complex series of cytokines, in particular tumour necrosis factor α (TNF-α), a proinflammatory mediator, and interleukin (IL)-10, an inhibitor of TNF-α, which downregulates granulomatous inflammation. A study of individuals with *S. haematobium* infection demonstrated that increased bladder-wall morbidity was associated with increased TNF-α production relative to IL-10 [94]. Cross-sectional surveys in endemic communities typically record egg counts increasing from the youngest age groups, reaching a peak in teenage years, followed by a decline across older age groups. [95]. It remains unclear to what extent the observed decline in egg counts across older age groups is due to behavioural changes or an acquired immune response, but there is evidence that protective immune responses in schistosomiasis do develop, albeit very slowly [95].

An individual’s immune response to schistosomiasis (and subsequent risk of morbidity as well as re-infection) is therefore likely determined by a combination of factors, which include age of first infection, infection intensity and duration. The fact all of these may shift following a programme of control presents challenges for projecting long-term morbidity profiles in populations.

Schistosome worm burdens in populations are generally overdispersed, with most people having light infections and few people having heavy intensity, corresponding to a low *k* value (§3). When fitting negative binomial distributions, which are often used in models of schistosome transmission, egg counts as a proxy for worm burden can inform estimates of heterogeneities in exposure. However, understanding the underlying human behaviours and ecological factors that create these heterogeneities is challenging. Water contact is assumed to decline with age [96,97], but additional factors such as occupational risk (for example, fishing), sex differences [98,99] and local variations in snail habitats [100] will contribute to defining the individual exposure parameter *s*_*i*_ in equation (3.2). Biases in participation in schistosomiasis programmes may contribute to increased heterogeneities in morbidity risk after interventions, and studies of social and economic factors that may determine non-participation in MDA programmes indicate alternative strategies may be necessary to ensure access to treatment, and therefore morbidity reduction, is equitable for at-risk populations [101,102].

#### (ii) Modelling morbidity

Mathematical models may play a role in projecting the schistosomiasis morbidity burden in a population, but first assumptions will need to be refined, with a need to both characterize and parameterize the ‘dose–response’ relationship between cumulative worm burden and risk of different morbidity outcomes, and evaluate how differences in immune response, which may contribute to morbidity risk, are distributed in endemic populations.

A key challenge for projecting the future morbidity burden associated with schistosomiasis is the wide range of morbidities associated with infection, and the fact that some morbidities are reversible while others are not. WHO thresholds relating to morbidity control and EPHP were originally based on analysis of correlations between egg counts and severe pathology [103,104]. These studies identified statistically significant relationships between heavily infected individuals and severe pathology, and at a time when praziquantel was not widely available, treatment guidance subsequently prioritized reducing heavy-intensity infections, with the assumption that if these infections could be eliminated, the most severe morbidity outcomes would likewise be eliminated. A more recent analysis which sought to re-evaluate these assumptions found morbidity metrics to be higher in schools with more than 5% prevalence of heavy intensity infection for both intestinal and urogenital forms of schistosomiasis, but also confirmed that the morbidity impact of light infections has been underestimated, with significant pathology seen both in light infections and after treatment [56,105].

As demonstrated in figure 2*e*,*f*, after an intervention it may be that morbidities associated with lower-intensity schistosome infection will come to represent a greater proportion of the disease burden in a population. For example, if threshold A2 corresponds to ‘heavy-intensity infection’ as per WHO guidelines, and A1 corresponds to low-intensity infections, reaching elimination targets may mean incidence of severe pathology associated with long-term heavy-intensity infections, such as periportal fibrosis, is minimal, but the morbidity associated with lower-intensity infections could represent an ongoing public health burden. Furthermore, thresholds for elimination as a public health problem do not currently correspond to interruption of transmission, and it is possible that, as with smoking, there is no minimal ‘safe’ level of exposure to schistosomes below which morbidity will not be seen (equivalent to A1 in figure 2), and eliminating all exposure in at-risk populations may be necessary to truly deliver on elimination of schistosomiasis-attributable morbidity.

### (c) Foodborne trematodiasis

The foodborne trematodiases are diseases caused by macroparasites with a common, foodborne, route of transmission and within the NTD framework encompass trematodes (flukes) from the genera *Opisthorchis*, *Clonorchis*, *Fasciola* and *Paragonimus* [3]. Here we limit our focus to the carcinogenic parasites *Opisthorchis viverrini* and *Chlonorchis sinensis*, which are estimated to infect around 10 million and 15 million people, respectively, across Southeast Asia and southern China [106,107]. The two parasites are closely related and share the same route of transmission, which involves two intermediate hosts: freshwater snails and cyprinid fish. Humans become infected by eating raw or undercooked fish encysted with infective metacercariae and, after ingestion, flukes migrate to the liver, where they reach maturity in the biliary tract.

Control of liver flukes has followed a different route from many other NTDs, with a stronger emphasis on changing the eating behaviour of communities that consume raw fish, in addition to treatment of diagnosed positive cases with the anthelmintic praziquantel [108]. The epidemiology of *O. viverrini* is best characterized in Thailand, where national surveys report an overall declining prevalence since 1980 [109], with high infection intensities persisting in spatially focal ‘hotspots’ [106]. It is unclear whether the prevalence of *O. viverrini* in other endemic countries, such as Lao PDR, Cambodia and Myanmar, is stable, increasing or decreasing at a national level [110,111]. Long-term survey data for *C. sinensis* in Guangdong province, China reports a substantial decline in prevalence from 65% in 1988–1992; however, the prevalence has remained broadly stable in the period 1998–2021 and remains more than 10% in certain localities [112].

Since the report by the International Agency for Research on Cancer (IARC) in 1994, *O. viverrini* and *C. sinensis* have been recognized as two of only eleven pathogens that directly cause human cancers [113]. Both *O. viverrini* and *C. sinensis* cause chronic pathology of the bile duct, which progresses from inflammation to periductal fibrosis, then bile duct thickening and dilation, and finally can lead to tumorigenesis in around 1% of infected people [114]. Adult flukes are thought to induce pathology in a number of ways, including through mechanical damage, autoimmune inflammatory responses and secreted products [115]. Some of the strongest evidence for parasite-mediated pathology relates to the production of granulin, a wound-healing protein. When gene expression of granulin is knocked down in *O. viverrini*, this results in significantly less morbidity in infected hamsters [116]. Much of the research to establish the carcinogenic potential of these parasites has been performed on *in vivo* models [115], whereas the onset and progression of fluke-induced cholangiocarcinoma in humans is less well characterized [117,118].

#### (i) Heterogeneity in exposure

Owing to the foodborne route of transmission, patterns of infection with *O. viverrini* and *C. sinensis* are driven by (i) consumption of raw or lightly fermented freshwater fish and (ii) the local density of metacercariae in fish populations. The expected number of worms acquired by a definitive host within a period of time, also known as the force of infection (see §3), can therefore be decomposed into two main components *λ*_*ij*_(*a*, *t*) = *γ*_*i*_(*a*)*Z*_*j*_(*t*); where *γ*_*i*_(*a*) is the feeding rate of susceptible fish for individual *i* and *Z*_*j*_(*t*) is the mean count of metacercariae in susceptible fish for locality *j*. Therefore, the variation in liver fluke infection intensity observed in epidemiological surveys is caused by variability in both human behavioural factors that influence diet, such as sex, ethnicity and socio-economic status [119,120], and environmental factors that underlie parasite density in the intermediate fish host [121].

The feeding rate of communities at risk of infection with liver flukes has not been observed over long periods. Evidence from fisheries suggests that the annual *per capita* consumption of fish is 27.2 kg in Thailand and 24.5 kg in Lao PDR [122,123]; however, national averages fail to capture variability in patterns of consumption within and between communities. Consumption of raw fish has been discouraged by public health campaigns in endemic areas for decades [108], yet it remains a culturally significant activity linked with regional identity and can be perceived to have health benefits, particularly for men [124]. Studies examining *O. viverrini* metacercariae in cyprinid fish have shown high variability in the number of metacercariae by fish species and also by season [125,126]. Studies on the ecological stages of the life cycle are rarely linked with epidemiological surveys [127], but a combined approach that also incorporates parasite genomic data would provide substantial insights into the geographic sources of liver fluke transmission.

#### (ii) Heterogeneity in pathological outcomes

Infection with both *O. viverrini* and *C. sinensis* has been repeatedly found in cross-sectional studies to be a risk factor for the development of liver pathology and cholangiocarcinoma [128,129], with higher infection intensities producing stronger associations with chronic disease. Haswell-Elkins and colleagues reporting elevated odds ratios of 1.7, 3.2 and 14 for individuals with 1–1500, 1501–6000 and more than 6000 *O. viverrini* eggs per gram of stool respectively in northeast Thailand [130]. While the categorization of egg count intensities varies by study, making direct comparisons difficult, subsequent surveys have reported similar strengths of association [131,132]. Similarly for *C. sinensis*, prevalence of the parasite is correlated with the incidence of cholangiocarcinoma at the population level [133], and infection intensity (eggs per gram of stool) is correlated with disability weight score (value for the severity of a condition, which are added together in the case of multiple sequelae) at the individual level [134]. Liver pathology in humans living in endemic settings is more prevalent in older age groups (more than 50 years), while infection with liver fluke can occur early in childhood [135], indicating a lag between the start of infection and the onset of liver disease. Taking these points together, it can be assumed that chronic damage to the bile ducts accumulates primarily as a function of the length of parasite exposure and intensity of infection [115].

As with many helminths, infection with liver flukes results in highly variable morbidity outcomes. A cohort study in Thailand found that of 3359 egg positive individuals for *O. viverrini*, 785 (23%) had severe periductal fibrosis as diagnosed by ultrasonography and four had a biliary mass indicative of cholangiocarcinoma (0.1%) [136]. Over five subsequent years of follow-up, five further individuals (who had tested positive for *O. viverrini* and periductal fibrosis at baseline) developed a liver mass, giving an incidence of cholangiocarcinoma in this population as approximately one case per 420 person-years. Similarly, a study on *C. sinensis* morbidity in Guangdong province, China found that of 259 egg-positive individuals with no parasitic co-infections, 92 (36%) had at least one sequela linked with clonorchiasis [134].

#### (iii) Modelling morbidity

Limited research has been performed on the population dynamics of liver fluke [47]. The first mathematical model for *O. viverrini* was published in 2018 [137], using a framework influenced by earlier macroparasite models, and this was subsequently extended to account for the impact of interventions with a focus on southern Lao PDR [138]. Similarly, a transmission model for *C. sinensis* in southern China was published in 2018 [139]; however, this compartmental model does not consider the worm burden, rather that hosts are either susceptible or infected, and inferred a large number of initial states and parameters from sparse data, leading to biologically implausible output (for instance, the initial population size of snails is 1026, whereas that of fish is 10.2 million). As with many other parasite transmission models, none of these studies considered parasite-induced pathology or elevated mortality in infected humans. Given high mortality rates from cholangiocarcinoma in liver fluke-endemic populations [128,140], this is a notable omission as elevated mortality in heavily infected individuals is likely a contributing factor to the lower mean worm burdens observed for older age groups (more than 50 years) in epidemiological surveys [135,141,142].

Making a quantitative link between cumulative worm burden and the probability of developing cholangiocarcinoma and other liver pathology remains a challenge. A potential method to estimate the latent period between fluke infection and tumorigenesis is provided by computational models that analyse the pattern of mutation in cancer genomes to infer the sequence of somatic mutations [143]. Given the early onset of mutations in the *TP53* tumour suppressor gene across a broad spectrum of human cancers [143] and a more than 80% occurrence of these mutations in fluke-associated chloangiocarcinoma cases [118], inferring the age at which driver mutations occur in the *TP53* gene is likely to be an important factor for understanding the latency period of fluke-induced cholangiocarcinoma and developing early prognosis tools for screening at-risk patients.

## 5. Conclusion

Public health interventions over recent years have been widely successful in reducing transmission and prevalence of the infectious agents associated with many NTDs, but for sequelae with a long-time course, reaching targets based on prevalence of infection may not necessarily be the end of morbidity risk for some individuals. Characterizing and quantifying the relationships between exposure, individual host factors and risk of long-term morbidity outcomes are critical to making projections that incorporate future morbidity burden for NTDs, and in some cases significant gaps in our understanding of these relationships remain. A further challenge is incorporating changes in the distribution of morbidity risk in populations over the course of, and after, an intervention, and establishing the impact this will have on prevalence and incidence of clinical outcomes. While changes in variables such as age and cumulative exposure over time can largely be captured by individual-based models, the impact of factors such as variations in immune responses by age at first exposure, which is likely to shift after an intervention, is often poorly understood. The role of individual genetic predisposition is largely unknown for many NTDs, but future work such as genomic studies and data on immunological markers may provide insights on the distribution of individual-level host risk factors in populations, which could be incorporated into transmission models. Post-intervention, there may be a shift in the relative proportion of the total morbidity burden attributable to different disease outcomes, and incorporating overarching morbidity metrics such as disability-adjusted life-years (DALYs) or quality-adjusted life-years (QALYs) to allow weighting of different sequelae should be an additional focus for future predictive model frameworks. However, some of the methodologies and assumptions involved with attributing such metrics have been critiqued [144–146], and further work may be needed to improve the applicability of existing metrics to NTDs. By providing quantitative insights on the dynamics of morbidity burden in the context of near-elimination and post-elimination dynamics, modelling approaches may play a key role in addressing challenges such as where morbidity interventions or screening (and associated funding) may be needed, for how long, and in which subsets of formerly endemic populations, questions of critical importance if control efforts are to truly eliminate the burden of morbidity associated with NTDs for all.

## Data Availability

This article has no additional data.

## References

[1] Hotez PJ, Fenwick A, Savioli L, Molyneux DH. 2009 Rescuing the bottom billion through control of neglected tropical diseases. Lancet **373**, 1570-1575. (10.1016/S0140-6736(09)60233-6) 19410718

[2] Molyneux DH. 2008 Combating the ‘other diseases’ of MDG 6: changing the paradigm to achieve equity and poverty reduction? Trans. R Soc. Trop. Med. Hyg. **102**, 509-519. (10.1016/j.trstmh.2008.02.024) 18413278

[3] WHO. 2021 *Ending the neglect to attain the sustainable development goals: a sustainability framework for action against neglected tropical diseases 2021–2030.* Geneva, Switzerland: World Health Organization. See https://apps.who.int/iris/handle/10665/338886.

[4] Hotez PJ. 2021 Forgotten people, forgotten diseases: the neglected tropical diseases and their impact on global health and development. New York, NY: John Wiley & Sons.

[5] Chesnais CB, Nana-Djeunga HC, Njamnshi AK, Lenou-Nanga CG, Boullé C, Bissek ACZK, Kamgno J, Colebunders R, Boussinesq M. 2018 The temporal relationship between onchocerciasis and epilepsy: a population-based cohort study. Lancet Infect. Dis. **18**, 1278-1286. (10.1016/S1473-3099(18)30425-0) 30268645

[6] Medley G, Bundy D. 1996 Dynamic modeling of epidemiologic patterns of schistosomiasis morbidity. Am. J. Trop. Med. Hyg. **55**, 149-158. (10.4269/ajtmh.1996.55.149) 8940969

[7] Gambhir M et al. 2009 The development of an age-structured model for trachoma transmission dynamics, pathogenesis and control. PLoS Negl. Trop. Dis. **3**, e462. (10.1371/journal.pntd.0000462) 19529762 PMC2691478

[8] Warren KS. 1978 The pathology, pathobiology and pathogenesis of schistosomiasis. Nature **273**, 609-612. (10.1038/273609a0) 351411

[9] Sithithaworn P, Haswell-Elkins MR, Mairiang P, Satarug S, Mairiang E, Vatanasapt V, Elkins DB. 1994 Parasite-associated morbidity: liver fluke infection and bile duct cancer in northeast Thailand. Int. J. Parasitol. **24**, 833-843. (10.1016/0020-7519(94)90009-4) 7982745

[10] Quinnell RJ. 2003 Genetics of susceptibility to human helminth infection. Int. J. Parasitol. **33**, 1219-1231. (10.1016/S0020-7519(03)00175-9) 13678637

[11] Mangano V, Modiano D. 2014 Host genetics and parasitic infections. Clin. Microbiol. Infect. **20**, 1265-1275. (10.1111/1469-0691.12793) 25273270

[12] Mawa PA, Kincaid-Smith J, Tukahebwa EM, Webster JP, Wilson S. 2021 Schistosomiasis morbidity hotspots: roles of the human host, the parasite and their interface in the development of severe morbidity. Front. Immunol. **12**, 635869. (10.3389/fimmu.2021.635869) 33790908 PMC8005546

[13] Wiegand RE et al. 2022 Defining elimination as a public health problem for schistosomiasis control programmes: beyond prevalence of heavy-intensity infections. Lancet Glob. Health **10**, e1355-e1359. (10.1016/S2214-109X(22)00287-X) 35961358 PMC10184143

[14] Truscott JE, Werkman M, Wright JE, Farrell SH, Sarkar R, Ásbjörnsdóttir K, Anderson RM. 2017 Identifying optimal threshold statistics for elimination of hookworm using a stochastic simulation model. Parasites Vectors **10**, 132. (10.1186/s13071-017-2256-8) 28666452 PMC5493114

[15] Hollingsworth TD et al. 2015 Quantitative analyses and modelling to support achievement of the 2020 goals for nine neglected tropical diseases. Parasites Vectors **8**, 630. (10.1186/s13071-015-1235-1) 26652272 PMC4674954

[16] Huang CI, Crump RE, Brown PE, Spencer SE, Miaka EM, Shampa C, Keeling MJ, Rock KS. 2022 Identifying regions for enhanced control of *gambiense* sleeping sickness in the Democratic Republic of Congo. Nat. Commun. **13**, 1448. (10.1038/s41467-022-29192-w) 35304479 PMC8933483

[17] Le Rutte EA et al. 2018 Policy recommendations from transmission modeling for the elimination of visceral leishmaniasis in the Indian subcontinent. Clin. Infect. Dis. **66**, S301-S308. (10.1093/cid/ciy007) 29860292 PMC5982727

[18] Antillon M, Huang CI, Crump RE, Brown PE, Snijders R, Miaka EM, Keeling MJ, Rock KS, Tediosi F. 2022 Cost-effectiveness of sleeping sickness elimination campaigns in five settings of the Democratic Republic of Congo. Nat. Commun. **13**, 1051. (10.1038/s41467-022-28598-w) 35217656 PMC8881616

[19] Blok DJ, De Vlas SJ, Richardus JH. 2015 Global elimination of leprosy by 2020: are we on track? Parasites Vectors **8**, 548. (10.1186/s13071-015-1143-4) 26490878 PMC4618543

[20] Blok DJ, Geluk A, Richardus JH. 2018 Minimum requirements and optimal testing strategies of a diagnostic test for leprosy as a tool towards zero transmission: a modeling study. PLoS Negl. Trop. Dis. **12**, e0006529. (10.1371/journal.pntd.0006529) 29799844 PMC5991769

[21] Turner HC, Walker M, Churcher TS, Basáñez MG. 2014 Modelling the impact of ivermectin on river blindness and its burden of morbidity and mortality in African savannah: EpiOncho projections. Parasites Vectors **7**, 241. (10.1186/1756-3305-7-241) 24886747 PMC4037555

[22] Coffeng LE et al. 2013 African programme for onchocerciasis control 1995–2015: model-estimated health impact and cost. PLoS Negl. Trop. Dis. **7**, e2032. (10.1371/journal.pntd.0002032) 23383355 PMC3561133

[23] VinkelesMelchers NVS, Stolk WA, Pedrique B, Bakker R, Murdoch ME, de Vlas SJ, Coffeng LE. 2021 The burden of skin disease and eye disease due to onchocerciasis in countries formerly under the African Programme for Onchocerciasis Control mandate for 1990, 2020, and 2030. PLoS Negl. Trop. Dis. **15**, e00096041. (10.1371/journal.pntd.0009604) PMC8312930 34310602

[24] Vinkeles Melchers NV, Stolk WA, Murdoch ME, Pedrique B, Kloek M, Bakker R, de Vlas SJ, Coffeng LE. 2021 How does onchocerciasis-related skin and eye disease in Africa depend on cumulative exposure to infection and mass treatment? PLoS Negl. Trop. Dis. **15**, e0009489. (10.1371/journal.pntd.0009489) 34115752 PMC8221783

[25] Graham M, Ayabina D, Lucas TC, Collyer BS, Medley GF, Hollingsworth TD, Toor J. 2021 SCHISTOX: an individual based model for the epidemiology and control of schistosomiasis. Infect. Dis. Modell. **6**, 438-447. (10.1016/j.idm.2021.01.010) PMC7897994 33665519

[26] Borlase A et al. 2021 Modelling trachoma post-2020: opportunities for mitigating the impact of COVID-19 and accelerating progress towards elimination. Trans. R Soc. Trop. Med. Hyg. **115**, 213-221. (10.1093/trstmh/traa171) 33596317 PMC7928577

[27] Medley G, Guyatt H, Bundy D. 1993 A quantitative framework for evaluating the effect of community treatment on the morbidity due to ascariasis. Parasitology **106**, 211-221. (10.1017/S0031182000075016) 8446474

[28] de Vlas SJ et al. 2016 Concerted efforts to control or eliminate neglected tropical diseases: how much health will be gained? PLoS Negl. Trop. Dis. **10**, e0004386. (10.1371/journal.pntd.0004386) 26890362 PMC4758649

[29] Rothman KJ. 1981 Induction and latent periods. Am. J. Epidemiol. **114**, 253-259. (10.1093/oxfordjournals.aje.a113189) 7304560

[30] Tammemägi MC et al. 2013 Selection criteria for lung-cancer screening. N. Engl. J. Med. **368**, 728-736. (10.1056/NEJMoa1211776) 23425165 PMC3929969

[31] Ruano-Ravina A, Figueiras A, Montes-Martínez A, Barros-Dios JM. 2003 Dose-response relationship between tobacco and lung cancer: new findings. Eur. J. Cancer Prevent. **12**, 257-263. (10.1097/00008469-200308000-00003) 12883376

[32] Zhang P, Chen PL, Li ZH, Zhang A, Zhang XR, Zhang YJ, Liu D, Mao C. 2022 Association of smoking and polygenic risk with the incidence of lung cancer: a prospective cohort study. Br. J. Cancer **126**, 1637-1646. (10.1038/s41416-022-01736-3) 35194190 PMC9130319

[33] World Health Organization. 2022 *WHO tobacco factsheet.* See https://www.who.int/docs/default-source/campaigns-and-initiatives/world-no-tobacco-day-2020/wntd-tobacco-fact-sheet.pdf (accessed 31 July 2023).

[34] Andrade-Mogrovejo DA et al. 2022 Development of a dose-response model for porcine cysticercosis. PLoS ONE **17**, e0264898. (10.1371/journal.pone.0264898) 35286329 PMC8920259

[35] Little M, Breitling L, Basáñez M, Alley E, Boatin B. 2004 Association between microfilarial load and excess mortality in onchocerciasis: an epidemiological study. Lancet **363**, 1514-1521. (10.1016/S0140-6736(04)16151-5) 15135599

[36] Walker M, Little MP, Wagner KS, Soumbey-Alley EW, Boatin BA, Basáñez MG. 2012 Density-dependent mortality of the human host in onchocerciasis: relationships between microfilarial load and excess mortality. PLoS Negl. Trop. Dis. **6**, e1578. (10.1371/journal.pntd.0001578) 22479660 PMC3313942

[37] Basáñez MG, Walker M, Turner H, Coffeng L, de Vlas S, Stolk W. 2016 River blindness: mathematical models for control and elimination. Adv. Parasitol. **94**, 247-341. (10.1016/bs.apar.2016.08.003) 27756456

[38] Pearl J, Mackenzie D. 2018 The book of why: the new science of cause and effect. New York, NY: Basic Books.

[39] Chanock SJ, Hunter DJ. 2008 When the smoke clears. Nature **452**, 537-538. (10.1038/452537a) 18385720

[40] Li Y et al. 2010 Genetic variants and risk of lung cancer in never smokers: a genome-wide association study. Lancet Oncol. **11**, 321-330. (10.1016/S1470-2045(10)70042-5) 20304703 PMC2945218

[41] Ganusov VV, Bergstrom CT, Antia R. 2002 Within-host population dynamics and the evolution of microparasites in a heterogeneous host population. Evolution **56**, 213-223. (10.1111/j.0014-3820.2002.tb01332.x) 11926490

[42] Sakthianandeswaren A, Foote SJ, Handman E. 2009 The role of host genetics in leishmaniasis. Trends Parasitol. **25**, 383-391. (10.1016/j.pt.2009.05.004) 19617002

[43] Woolhouse ME et al. 2015 Co-infections determine patterns of mortality in a population exposed to parasite infection. Sci. Adv. **1**, e1400026. (10.1126/sciadv.1400026) 26601143 PMC4643819

[44] Anderson RM, May RM. 1991 Infectious diseases of humans: dynamics and control. Oxford, UK: Oxford University Press.

[45] Crellen T, Haswell M, Sithithaworn P, Sayasone S, Odermatt P, Lamberton PH, Spencer SE, Hollingsworth TD. 2023 Diagnosis of helminths depends on worm fecundity and the distribution of parasites within hosts. Proc. R. Soc. B **290**, 20222204. (10.1098/rspb.2022.2204) PMC9845982 36651047

[46] Vineis P et al. 2004 Tobacco and cancer: recent epidemiological evidence. J. Natl Cancer Inst. **96**, 99-106. (10.1093/jnci/djh014) 14734699

[47] Crellen T, Sithithaworn P, Pitaksakulrat O, Khuntikeo N, Medley GF, Hollingsworth TD. 2021 Towards evidence-based control of *Opisthorchis viverrini*. Trends Parasitol. **37**, 370-380. (10.1016/j.pt.2020.12.007) 33516657

[48] Walker M, Hall A, Basáñez MG. 2013 *Ascaris lumbricoides*: new epidemiological insights and mathematical approaches. In *Ascaris: the neglected parasite* (ed. C Holland), pp. 155–201. Amsterdam, The Netherlands: Elsevier.

[49] Bolker BM. 2008 Ecological models and data in R. Princeton, NJ: Princeton University Press.

[50] Gelman A, Carlin JB, Stern HS, Dunson DB, Vehtari A, Rubin DB. 2013 Bayesian data analysis. Boca Raton, FL: CRC Press.

[51] Wilson K, Bjørnstad O, Dobson A, Merler S, Poglayen G, Randolph S, Read A, Skorping A. 2002 Heterogeneities in macroparasite infections: patterns and processes. In *The ecology of wildlife diseases* (eds JP Hudson, A Rizzoli, BT Grenfell, H Heesterbeek, AP Dobson), pp. 6–44. Oxford, UK: Oxford University Press.

[52] Chan M, Guyatt H, Bundy D, Medley G. 1996 Dynamic models of schistosomiasis morbidity. Am. J. Trop. Med. Hyg. **55**, 52-62. (10.4269/ajtmh.1996.55.52) 8702023

[53] Chan M, Anderson R, Medley G, Bundy D. 1996 Dynamic aspects of morbidity and acquired immunity in schistosomiasis control. Acta Trop. **62**, 105-117. (10.1016/S0001-706X(96)00039-3) 8988311

[54] de Vlas S, Gryseels B, Van Oortmarssen G, Polderman A, Habbema J. 1992 A model for variations in single and repeated egg counts in *Schistosoma mansoni* infections. Parasitology **104**, 451-460. (10.1017/S003118200006371X) 1641245

[55] Sriamporn S, Pisani P, Pipitgool V, Suwanrungruang K, Kamsa-Ard S, Parkin D. 2004 Prevalence of *Opisthorchis viverrini* infection and incidence of cholangiocarcinoma in Khon Kaen, northeast Thailand. Trop. Med. Int. Health **9**, 588-594. (10.1111/j.1365-3156.2004.01234.x) 15117303

[56] Wiegand RE et al. 2021 Associations between infection intensity categories and morbidity prevalence in school-age children are much stronger for *Schistosoma haematobium* than for *S. mansoni*. PLoS Negl. Trop. Dis. **15**, e0009444. (10.1371/journal.pntd.0009444) 34033646 PMC8183985

[57] Diggle PJ. 2011 Estimating prevalence using an imperfect test. Epidemiol. Res. Int. **2011**, 608719. (10.1155/2011/608719)

[58] van der Werf MJ, de Vlas SJ, Brooker S, Looman CW, Nagelkerke NJ, Habbema JDF, Engels D. 2003 Quantification of clinical morbidity associated with schistosome infection in sub-Saharan Africa. Acta Trop. **86**, 125-139. (10.1016/S0001-706X(03)00029-9) 12745133

[59] van der Werf M, de Vlas S. 2004 Diagnosis of urinary schistosomiasis: a novel approach to compare bladder pathology measured by ultrasound and three methods for hematuria detection. Am. J. Trop. Med. Hyg. **71**, 98-106. (10.4269/ajtmh.2004.71.98) 15238697

[60] Wiegand RE, Fleming FM, Straily A, Montgomery SP, de Vlas SJ, Utzinger J, Vounatsou P, Secor WE. 2021 Urogenital schistosomiasis infection prevalence targets to determine elimination as a public health problem based on microhematuria prevalence in school-age children. PLoS Negl. Trop. Dis. **15**, e0009451. (10.1371/journal.pntd.0009451) 34115760 PMC8221785

[61] Prada JM, Touloupou P, Adriko M, Tukahebwa EM, Lamberton PH, Hollingsworth TD. 2018 Understanding the relationship between egg- and antigen-based diagnostics of *Schistosoma mansoni* infection pre- and post-treatment in Uganda. Parasites Vectors **11**, 21. (10.1186/s13071-017-2580-z) 29310695 PMC5759883

[62] Solomon AW, Burton MJ, Gower EW, Harding-Esch EM, Oldenburg CE, Taylor HR, Traoré L. 2022 Trachoma. Nat. Rev. Dis. Primers **8**, 32. (10.1038/s41572-022-00359-5) 35618795

[63] World Health Organization. 2022 *WHO trachoma factsheet*. See https://www.who.int/news-room/fact-sheets/detail/trachoma (accessed 31/7/2023).

[64] Burton MJ, Mabey DCW. 2009 The global burden of trachoma: a review. PLoS Negl. Trop. Dis. **3**, e460. (10.1371/journal.pntd.0000460) 19859534 PMC2761540

[65] Munoz B, Aron J, Turner V, West S. 1997 Incidence estimates of late stages of trachoma among women in a hyperendemic area of central Tanzania. Trop. Med. Int. Health **2**, 1030-1038. (10.1046/j.1365-3156.1997.d01-186.x) 9391505

[66] Astale T et al. 2021 The population-based prevalence of trachomatous scarring in a trachoma hyperendemic setting: results from 152 impact surveys in Amhara, Ethiopia. BMC Ophthalmol. **21**, 213. (10.1186/s12886-021-01972-w) 33985443 PMC8120834

[67] Wolle MA, Muñoz B, Mkocha H, West SK. 2009 Age, sex, and cohort effects in a longitudinal study of trachomatous scarring. Invest. Ophthalmol. Vis. Sci. **50**, 592-596. (10.1167/iovs.08-2414) 18936137 PMC3820011

[68] West SK, Muñoz B, Mkocha H, Hsieh YH, Lynch MC. 2001 Progression of active trachoma to scarring in a cohort of Tanzanian children. Ophthal. Epidemiol. **8**, 137-144. (10.1076/opep.8.2.137.4158) 11471083

[69] Burton MJ et al. 2015 Pathogenesis of progressive scarring trachoma in Ethiopia and Tanzania and its implications for disease control: two cohort studies. PLoS Negl. Trop. Dis. **9**, e0003763. (10.1371/journal.pntd.0003763) 25970613 PMC4430253

[70] Ramadhani AM et al. 2019 Progression of scarring trachoma in Tanzanian children: a four-year cohort study. PLoS Negl. Trop. Dis. **13**, e0007638. (10.1371/journal.pntd.0007638) 31412025 PMC6709924

[71] Last A et al. 2017 Spatial clustering of high load ocular *Chlamydia trachomatis* infection in trachoma: a cross-sectional population-based study. Pathogens Dis. **75**, ftx050. (10.1093/femspd/ftx050) PMC5808645 28472466

[72] Bailey R, Osmond C, Mabey DC, Whittle HC, Ward ME. 1989 Analysis of the household distribution of trachoma in a Gambian village using a Monte Carlo simulation procedure. Int. J. Epidemiol. **18**, 944-951. (10.1093/ije/18.4.944) 2621031

[73] Polack SR, Solomon AW, Alexander ND, Massae PA, Safari S, Shao JF, Foster A, Mabey DC. 2005 The household distribution of trachoma in a Tanzanian village: an application of GIS to the study of trachoma. Trans. R Soc. Trop. Med. Hyg. **99**, 218-225. (10.1016/j.trstmh.2004.06.010) 15653125 PMC6917506

[74] Habtamu E et al. 2015 Trachoma and relative poverty: a case-control study. PLoS Negl. Trop. Dis. **9**, e0004228. (10.1371/journal.pntd.0004228) 26600211 PMC4657919

[75] Courtright P, West SK. 2004 Contribution of sex-linked biology and gender roles to disparities with trachoma. Emerg. Infect. Dis. **10**, 2012-2016. (10.3201/eid1011.040353) 15550216 PMC3328994

[76] Berry A, Hall JV. 2019 The complexity of interactions between female sex hormones and *Chlamydia trachomatis* infections. Curr. Clin. Microbiol. Rep. **6**, 67-75. (10.1007/s40588-019-00116-5) 31890462 PMC6936955

[77] Bailey R, Duong T, Carpenter R, Whittle H, Mabey D. 1999 The duration of human ocular *Chlamydia trachomatis* infection is age dependent. Epidemiol. Infect. **123**, 479-486. (10.1017/s0950268899003076) 10694161 PMC2810784

[78] Grassly NC, Ward ME, Ferris S, Mabey DC, Bailey RL. 2008 The natural history of trachoma infection and disease in a Gambian cohort with frequent follow-up. PLoS Negl. Trop. Dis. **2**, e341. (10.1371/journal.pntd.0000341) 19048024 PMC2584235

[79] Hu VH, Holland MJ, Burton MJ. 2013 Trachoma: protective and pathogenic ocular immune responses to *Chlamydia trachomatis*. PLoS Negl. Trop. Dis. **7**, e2020. (10.1371/journal.pntd.0002020) 23457650 PMC3573101

[80] Lietman TM, Oldenburg CE, Keenan JD. 2020 Trachoma: time to talk eradication. Ophthalmology **127**, 11-13. (10.1016/j.ophtha.2019.11.001) 31864470

[81] Amza A et al. 2013 The easiest children to reach are most likely to be infected with ocular *Chlamydia trachomatis* in trachoma endemic areas of Niger. PLoS Negl. Trop. Dis. **7**, e1983. (10.1371/journal.pntd.0001983) 23326612 PMC3542188

[82] Khandekar R, Mohammed AJ. 2007 The prevalence of trachomatous trichiasis in Oman (Oman Eye Study 2005). Ophthal. Epidemiol. **14**, 267-272. (10.1080/09286580601160622) 17994435

[83] Bowman RJ, Jatta B, Cham B, Bailey RL, Faal H, Myatt M, Foster A, Johnson GJ. 2001 Natural history of trachomatous scarring in The Gambia: results of a 12-year longitudinal follow-up. Ophthalmology **108**, 2219-2224. (10.1016/s0161-6420(01)00645-5) 11733262

[84] Colley DG, Bustinduy AL, Secor WE, King CH. 2014 Human schistosomiasis. Lancet **383**, 2253-2264. (10.1016/S0140-6736(13)61949-2) 24698483 PMC4672382

[85] Santos LL, Santos J, Gouveia MJ, Bernardo C, Lopes C, Rinaldi G, Brindley PJ. 2021 Urogenital schistosomiasis—history, pathogenesis, and bladder cancer. J. Clin. Med. **10**, 205. (10.3390/jcm10020205) 33429985 PMC7826813

[86] Ishida K, Hsieh MH. 2018 Understanding urogenital schistosomiasis-related bladder cancer: an update. Front. Med. **5**, 223. (10.3389/fmed.2018.00223) PMC6104441 30159314

[87] Hotez PJ, Fenwick A. 2009 Schistosomiasis in Africa: an emerging tragedy in our new global health decade. PLoS Negl. Trop. Dis. **3**, e485. (10.1371/journal.pntd.0000485) 19787054 PMC2746322

[88] Kokaliaris C et al. 2022 Effect of preventive chemotherapy with praziquantel on schistosomiasis among school-aged children in sub-Saharan Africa: a spatiotemporal modelling study. Lancet Infect. Dis. **22**, 136-149. (10.1016/S1473-3099(21)00090-6) 34863336 PMC8695385

[89] McManus DP, Dunne DW, Sacko M, Utzinger J, Vennervald BJ, Zhou XN. 2018 Schistosomiasis. Nat. Rev. Dis. Primers **4**, 13. (10.1038/s41572-018-0013-8) 30093684

[90] Ross AG, Vickers D, Olds GR, Shah SM, McManus DP. 2007 Katayama syndrome. Lancet. Infect. Dis. **7**, 218-224. (10.1016/S1473-3099(07)70053-1) 17317603

[91] Fallon PG, Richardson EJ, Smith P, Dunne DW. 2000 Elevated type 1, diminished type 2 cytokines and impaired antibody response are associated with hepatotoxicity and mortalities during *Schistosoma mansoni* infection of CD4-depleted mice. Eur. J. Immunol. **30**, 470-480. (10.1002/1521-4141(200002)30:2¡470::AID-IMMU470¿3.0.CO;2-T) 10671202

[92] Grogan JL, Kremsner PG, Deelder AM, Yazdanbakhsh M. 1998 Antigen-specific proliferation and interferon-gamma and interleukin-5 production are down-regulated during *Schistosoma haematobium* infection. J. Infect. Dis. **177**, 1433-1437. (10.1086/517832) 9593042

[93] King CL, Malhotra I, Jia X. 1996 *Schistosoma mansoni*: protective immunity in IL-4-deficient mice. Exp. Parasitol. **84**, 245-252. (10.1006/expr.1996.0110) 8932774

[94] Wamachi AN et al. 2004 Increased ratio of tumor necrosis factor-alpha to interleukin-10 production is associated with *Schistosoma haematobium*-induced urinary-tract morbidity. J. Infect. Dis. **190**, 2020-2030. (10.1086/425579) 15529268

[95] Kura K, Hardwick RJ, Truscott JE, Anderson RM. 2021 What is the impact of acquired immunity on the transmission of schistosomiasis and the efficacy of current and planned mass drug administration programmes? PLoS Negl. Trop. Dis. **15**, e0009946. (10.1371/journal.pntd.0009946) 34851952 PMC8635407

[96] Dalton PR, Pole D. 1978 Water-contact patterns in relation to *Schistosoma haematobium* infection. Bull. World Health Organ. **56**, 417-426. 308406 PMC2395583

[97] Barbour AD. 1985 The importance of age and water contact patterns in relation to *Schistosoma haematobium* infection. Trans. R. Soc. Trop. Med. Hyg. **79**, 151-153. (10.1016/0035-9203(85)90319-0) 4002283

[98] Pinot de Moira A, Fulford AJC, Kabatereine NB, Kazibwe F, Ouma JH, Dunne DW, Booth M. 2007 Microgeographical and tribal variations in water contact and *Schistosoma mansoni* exposure within a Ugandan fishing community. Trop. Med. Int. Health **12**, 724-735. (10.1111/j.1365-3156.2007.01842.x) 17550469

[99] Ayabina DV, Clark J, Bayley H, Lamberton PHL, Toor J, Hollingsworth TD. 2021 Gender-related differences in prevalence, intensity and associated risk factors of *Schistosoma* infections in Africa: a systematic review and meta-analysis. PLoS Negl. Trop. Dis. **15**, e0009083. (10.1371/journal.pntd.0009083) 34788280 PMC8635327

[100] Wood CL *et al.* 2019 Precision mapping of snail habitat provides a powerful indicator of human schistosomiasis transmission. Proc. Natl Acad. Sci. USA **116**, 23 182-23 191. (10.1073/pnas.1903698116) 31659025 PMC6859407

[101] Chami GF, Kontoleon AA, Bulte E, Fenwick A, Kabatereine NB, Tukahebwa EM, Dunne DW. 2016 Profiling nonrecipients of mass drug administration for schistosomiasis and hookworm infections: a comprehensive analysis of praziquantel and albendazole coverage in community-directed treatment in Uganda. Clin. Infect. Dis. **62**, 200-207. (10.1093/cid/civ829) 26409064 PMC4690482

[102] Chami GF, Bundy DAP. 2019 More medicines alone cannot ensure the treatment of neglected tropical diseases. Lancet Infect. Dis. **19**, e330-e336. (10.1016/S1473-3099(19)30160-4) 31160190

[103] WHO Expert Committee on Schistosomiasis Control, World Health Organization. 1973 *Schistosomiasis control: report of a WHO expert committee (meeting held in Geneva 3–7 July 1972)*. Geneva, Switzerland: World Health Organization. See https://apps.who.int/iris/handle/10665/41029.

[104] WHO. 1988 *Progress in assessment of morbidity due to Schistsoma mansoni infection: a review of recent literature.* Geneva, Switzerland: World Health Organization. See https://apps.who.int/iris/handle/10665/62416.

[105] King CH, Dickman K, Tisch DJ. 2005 Reassessment of the cost of chronic helmintic infection: a meta-analysis of disability-related outcomes in endemic schistosomiasis. Lancet **365**, 1561-1569. (10.1016/S0140-6736(05)66457-4) 15866310

[106] Suwannatrai A, Saichua P, Haswell M. 2018 Epidemiology of *Opisthorchis viverrini* infection. Adv. Parasitol. **101**, 41-67. (10.1016/bs.apar.2018.05.002) 29907255

[107] Na BK, Pak JH, Hong SJ. 2020 *Clonorchis sinensis* and clonorchiasis. Acta Trop. **203**, 105309. (10.1016/j.actatropica.2019.105309) 31862466

[108] Jongsuksuntigul P, Imsomboon T. 2003 Opisthorchiasis control in Thailand. Acta Trop. **88**, 229-232. (10.1016/j.actatropica.2003.01.002) 14611877

[109] Wattanawong O et al. 2021 Current status of helminthiases in Thailand: a cross-sectional, nationwide survey, 2019. Acta Trop. **223**, 106082. (10.1016/j.actatropica.2021.106082) 34364893

[110] Khieu V, Fürst T, Miyamoto K, Yong TS, Chai JY, Huy R, Muth S, Odermatt P. 2019 Is *Opisthorchis viverrini* emerging in Cambodia? Adv. Parasitol. **103**, 31-73. (10.1016/bs.apar.2019.02.002) 30878058

[111] Sripa B, Suwannatrai AT, Sayasone S, Do DT, Khieu V, Yang Y. 2021 Current status of human liver fluke infections in the Greater Mekong Subregion. Acta Trop. **224**, 106133. (10.1016/j.actatropica.2021.106133) 34509453

[112] Fang Y et al. 2022 Long-term trend analysis of major human helminth infections — Guangdong Province, China, 1988–2021. China CDC Wkly **4**, 912-919. (https://pubmed.ncbi.nlm.nih.gov/36426289/) 36426289 10.46234/ccdcw2022.188 PMC9681603

[113] IARC Working Group on the Evaluation of Carcinogenic Risks to Humans. 1994 *Schistosomes, liver flukes and Helicobacter pylori*. Lyon, France: International Agency for Research on Cancer. PMC7681621 7715068

[114] Chamadol N, Khuntikeo N, Thinkhamrop B, Thinkhamrop K, Suwannatrai AT, Kelly M, Promthet S. 2019 Association between periductal fibrosis and bile duct dilatation among a population at high risk of cholangiocarcinoma: a cross-sectional study of cholangiocarcinoma screening in northeast Thailand. BMJ Open **9**, e023217. (10.1136/bmjopen-2018-023217) PMC6475358 30898798

[115] Sripa B, Brindley PJ, Mulvenna J, Laha T, Smout MJ, Mairiang E, Bethony JM, Loukas A. 2012 The tumorigenic liver fluke *Opisthorchis viverrini* – multiple pathways to cancer. Trends Parasitol. **28**, 395-407. (10.1016/j.pt.2012.07.006) 22947297 PMC3682777

[116] Arunsan P et al. 2019 Programmed knockout mutation of liver fluke granulin attenuates virulence of infection-induced hepatobiliary morbidity. eLife **8**, e41463. (10.7554/eLife.41463) 30644359 PMC6355195

[117] Kim TS, Pak JH, Kim JB, Bahk YY. 2016 *Clonorchis sinensis*, an oriental liver fluke, as a human biological agent of cholangiocarcinoma: a brief review. BMB Rep. **49**, 590. (10.5483/BMBRep.2016.49.11.109) 27418285 PMC5346318

[118] Jusakul A. 2017 Whole-genome and epigenomic landscapes of etiologically distinct subtypes of cholangiocarcinomaintegrative genomic and epigenomic analysis of cholangiocarcinoma. Cancer Discov. **7**, 1116-1135. (10.1158/2159-8290.CD-17-0368) 28667006 PMC5628134

[119] Forrer A et al. 2012 Spatial distribution of, and risk factors for, *Opisthorchis viverrini* infection in southern Lao PDR. PLoS Negl. Trop. Dis. **6**, e1481. (10.1371/journal.pntd.0001481) 22348157 PMC3279336

[120] Vinh HQ et al. 2017 Risk factors for *Clonorchis sinensis* infection transmission in humans in northern Vietnam: a descriptive and social network analysis study. Parasitol. Int. **66**, 74-82. (10.1016/j.parint.2016.11.018) 27939296 PMC5292293

[121] Namsanor J, Kiatsopit N, Laha T, Andrews RH, Petney TN, Sithithaworn P. 2020 Infection dynamics of *Opisthorchis viverrini* metacercariae in cyprinid fishes from two endemic areas in Thailand and Lao PDR. Am. J. Trop. Med. Hyg. **102**, 110. (10.4269/ajtmh.19-0432) 31701859 PMC6947784

[122] FAO. 2019 Thailand. In *Fishery and aquaculture country profiles*. Rome, Italy: Fisheries and Aquaculture Department, Food and Agriculture Organization of the United Nations. See https://www.fao.org/fishery/en/collection/facp.

[123] Hortle KG. 2007 Consumption and the yield of fish and other aquatic animals from the Lower Mekong Basin. *Mekong River Commission Tech. Pap.*, no. 16

[124] Grundy-Warr C, Andrews RH, Sithithaworn P, Petney TN, Sripa B, Laithavewat L, Ziegler AD. 2012 Raw attitudes, wetland cultures, life-cycles: socio-cultural dynamics relating to *Opisthorchis viverrini* in the Mekong Basin. Parasitol. Int. **61**, 65-70. (10.1016/j.parint.2011.06.015) 21712097

[125] Sithithaworn P, Pipitgool V, Srisawangwong T, Elkins DB, Haswell-Elkins MR. 1997 Seasonal variation of *Opisthorchis viverrini* infection in cyprinoid fish in north-east Thailand: implications for parasite control and food safety. Bull. World Health Organ. **75**, 125. 9185364 PMC2486929

[126] Pinlaor S et al. 2013 Distribution and abundance of *Opisthorchis viverrini* metacercariae in cyprinid fish in northeastern Thailand. Korean J. Parasitol. **51**, 703. (10.3347/kjp.2013.51.6.703) 24516277 PMC3916461

[127] Charoensuk L, Ribas A, Chedtabud K, Prakobwong S. 2022 Infection rate of *Opisthorchis viverrini* metacercariae in cyprinoid fish from the markets and its association to human opisthorchiasis in the local community in the northeast Thailand. Acta Trop. **225**, 106216. (10.1016/j.actatropica.2021.106216) 34717889

[128] Parkin DM, Srivatanakul P, Khlat M, Chenvidhya D, Chotiwan P, Insiripong S, L’abbé KA, Wild CP. 1991 Liver cancer in Thailand. I. A case-control study of cholangiocarcinoma. Int. J. Cancer **48**, 323-328. (10.1002/ijc.2910480302) 1645697

[129] Choi D et al. 2006 Cholangiocarcinoma and *Clonorchis sinensis* infection: a case–control study in Korea. J. Hepatol. **44**, 1066-1073. (10.1016/j.jhep.2005.11.040) 16480786

[130] Haswell-Elkins MR, Mairiang E, Mairiang P, Chaiyakum J, Chamadol N, Loapaiboon V, Sithithaworn P, Elkins DB. 1994 Cross-sectional study of *Opisthorchis viverrini* infection and cholangiocarcinoma in communities within a high-risk area in northeast Thailand. Int. J. Cancer **59**, 505-509. (10.1002/ijc.2910590412) 7960220

[131] Honjo S et al. 2005 Genetic and environmental determinants of risk for cholangiocarcinoma via *Opisthorchis viverrini* in a densely infested area in Nakhon Phanom, northeast Thailand. Int. J. Cancer **117**, 854-860. (10.1002/ijc.21146) 15957169

[132] Thaewnongiew K, Singthong S, Kutchamart S, Tangsawad S, Promthet S, Sailugkum S, Wongba N. 2014 Prevalence and risk factors for *Opisthorchis viverrini* infections in upper northeast Thailand. Asian Pac. J. Cancer Prev. **15**, 6609-6612. (10.7314/APJCP.2014.15.16.6609) 25169496

[133] Lim MK et al. 2006 *Clonorchis sinensis* infection and increasing risk of cholangiocarcinoma in the Republic of Korea. *Am. J. Trop. Med. Hyg.* **75**, 93–96.

[134] Qian MB et al. 2011 Disability weight of *Clonorchis sinensis* infection: captured from community study and model simulation. PLoS Negl. Trop. Dis. **5**, e1377. (10.1371/journal.pntd.0001377) 22180791 PMC3236727

[135] Haswell-Elkins M, Elkins DB, Sithithaworn P, Treesarawat P, Kaewkes S. 1991 Distribution patterns of *Opisthorchis viverrini* within a human community. Parasitology **103**, 97-101. (10.1017/S0031182000059333) 1945529

[136] Mairiang E, Laha T, Kaewkes S, Loukas A, Bethony J, Brindley PJ, Sripa B. 2021 Hepatobiliary morbidities detected by ultrasonography in *Opisthorchis viverrini*-infected patients before and after praziquantel treatment: a five-year follow up study. Acta Trop. **217**, 105853. (10.1016/j.actatropica.2021.105853) 33548204 PMC9614731

[137] Bürli C, Harbrecht H, Odermatt P, Sayasone S, Chitnis N. 2018 Mathematical analysis of the transmission dynamics of the liver fluke, *Opisthorchis viverrini*. J. Theor. Biol. **439**, 181-194. (10.1016/j.jtbi.2017.11.020) 29197514

[138] Bürli C, Harbrecht H, Odermatt P, Sayasone S, Chitnis N. 2018 Analysis of interventions against the liver fluke, *Opisthorchis viverrini*. Math. Biosci. **303**, 115-125. (10.1016/j.mbs.2018.06.008) 29958977

[139] Yuan R, Huang J, Zhang X, Ruan S. 2018 Modeling the transmission dynamics of clonorchiasis in Foshan, China. Scient. Rep. **8**, 15176. (10.1038/s41598-018-33431-w) PMC6181966 30310106

[140] Kamsa-Ard S et al. 2019 Cholangiocarcinoma trends, incidence, and relative survival in Khon Kaen, Thailand from 1989 through 2013: a population-based cancer registry study. J. Epidemiol. **29**, 197-204. (10.2188/jea.JE20180007) 30078813 PMC6445798

[141] Sithithaworn P et al. 1991 Quantitative post-mortem study of *Opisthorchis viverrini* in man in north-east Thailand. Trans. R. Soc. Trop. Med. Hyg. **85**, 765-768. (10.1016/0035-9203(91)90449-9) 1801350

[142] Sato M et al. 2015 Patterns of trematode infections of *Opisthorchis viverrini* (Opisthorchiidae) and *Haplorchis taichui* (Heterophyidae) in human populations from two villages in Savannakhet Province, Lao PDR. J. Helminthol. **89**, 439-445. (10.1017/S0022149X14000261) 24739959

[143] Gerstung M et al. 2020 The evolutionary history of 2,658 cancers. Nature **578**, 122-128. (10.1038/s41586-019-1907-7) 32025013 PMC7054212

[144] King CH, Bertino AM. 2008 Asymmetries of poverty: why global burden of disease valuations underestimate the burden of neglected tropical diseases. PLoS Negl. Trop. Dis. **2**, e209. (10.1371/journal.pntd.0000209) 18365036 PMC2267491

[145] Voigt K, King NB. 2014 Disability weights in the global burden of disease 2010 study: two steps forward, one step back? Bull. World Health Org. **92**, 226-228. (10.2471/BLT.13.126227) 24700983 PMC3949595

[146] Herricks JR et al. 2017 The global burden of disease study 2013: what does it mean for the NTDs? PLoS Negl. Trop. Dis. **11**, e0005424. (10.1371/journal.pntd.0005424) 28771480 PMC5542388

